# Comparison of latent tuberculosis infection screening strategies before tumor necrosis factor inhibitor treatment in inflammatory arthritis: IGRA-alone versus combination of TST and IGRA

**DOI:** 10.1371/journal.pone.0198756

**Published:** 2018-07-05

**Authors:** Dae Hyun Jeong, Jieun Kang, Young Ju Jung, Bin Yoo, Chang-Keun Lee, Yong-Gil Kim, Seokchan Hong, Tae Sun Shim, Kyung-Wook Jo

**Affiliations:** 1 Division of Pulmonology and Critical Care Medicine, University of Ulsan College of Medicine, Asan Medical Center, Seoul, South Korea; 2 Department of Rheumatology, University of Ulsan College of Medicine, Asan Medical Center, Seoul, South Korea; Agencia de Salut Publica de Barcelona, SPAIN

## Abstract

This study aims to compare the latent tuberculosis infection (LTBI) screening strategy of interferon-gamma release assay (IGRA)-alone and in combination with tuberculin skin tests (TSTs) before the initiation of tumor necrosis factor (TNF) inhibitor treatment in patients with inflammatory arthritis. Between January 2011 and June 2017, we enrolled 476 patients who were followed up for ≥1 year after the TNF inhibitor initiation in a tertiary referral center in South Korea. Inflammatory arthritis comprised rheumatoid arthritis in 266 (55.9%) and ankylosing spondylitis in 210 (44.1%) patients. The following strategies were used for LTBI screening during the study period: (i) from January 2011 to October 2014, the combination of TST and QuantiFERON-TB Gold In-Tube (QFT-GIT); (ii) between November 2014 and February 2015, QFT-GIT-alone and (iii) since March 2015, either the combination of TST and QFT-GIT or QFT-GIT-alone depending on the attending physician's choice. We compared the screening strategies of QFT-GIT alone and in combination with TST. Overall, 338 (71.0%) patients received LTBI screening tests using the combination of TST and QFT-GIT, and 138 (29.0%) received QFT-GIT-alone. In addition, the LTBI tests were positive in 159 (47.0%) of 338 patients using the combination tests, and 43.8% (148/338) required LTBI treatment. Meanwhile, the LTBI tests were positive in 32.6% (45/138) of QFT-GIT-alone patients, and 30.4% (42/138) required LTBI treatment. Among 338 patients who received combination tests, 2 patients developed active tuberculosis within 1 year after the TNF inhibitor initiation. Of patients who received QFT-GIT-alone, no patient developed tuberculosis. In conclusion, among patients who received QFT-GIT-alone, the number of patients who required LTBI treatment declined compared to the TST and QFT-GIT combination, and none developed active tuberculosis within 1 year, suggesting that QFT-GIT-alone could be a potential screening strategy for diagnosing LTBI in patients with inflammatory arthritis in South Korea.

## Introduction

Tumor necrosis factor (TNF) inhibitor has been increasingly used in the treatment of inflammatory arthritis, including rheumatoid arthritis (RA) and ankylosing spondylitis (AS), with profound effect [1, 2]. However, patients treated with TNF inhibitors are at increased risk of developing tuberculosis, predominantly through the reactivation of latent tuberculosis infection (LTBI) [3]. Owing to the dramatic reduction of the incidence of tuberculosis because of widespread screening and treatment of LTBI before the initiation of TNF inhibitors [4], screening for LTBI before starting therapy with TNF inhibitor is recommended [5].

Current diagnostic tests for LTBI comprise tuberculin skin tests (TST) and/or the interferon-gamma release assay (IGRA). Regarding LTBI screening strategies that use TST and/or IGRA, however, recommendations from different professional organizations and countries are inconsistent with each other because both tests have their limitations, such as accuracy, false-positive or -negative results according to the bacillus Calmette–Guérin (BCG) vaccination history, immunosuppression, cost, and complexity [6–9]. That is, some guidelines recommend TST or IGRA only [10–12], whereas other guidelines recommend a combination of TST and IGRA before initiation of the TNF inhibitor [13, 14]. Nevertheless, the optimal methods for LTBI screening in patients with inflammatory arthritis remain controversial because no study, to date, has compared various strategies directly [9, 15].

Hence, this study aims to compare the screening strategies of IGRA-alone and in combination with TST before the initiation of a TNF inhibitor in patients with inflammatory arthritis in South Korea, where the incidence of tuberculosis is intermediate (80–100/100,000 per year) and BCG vaccination is mandatory at birth [16, 17].

## Materials and methods

### Study subjects

**Fig 1:**
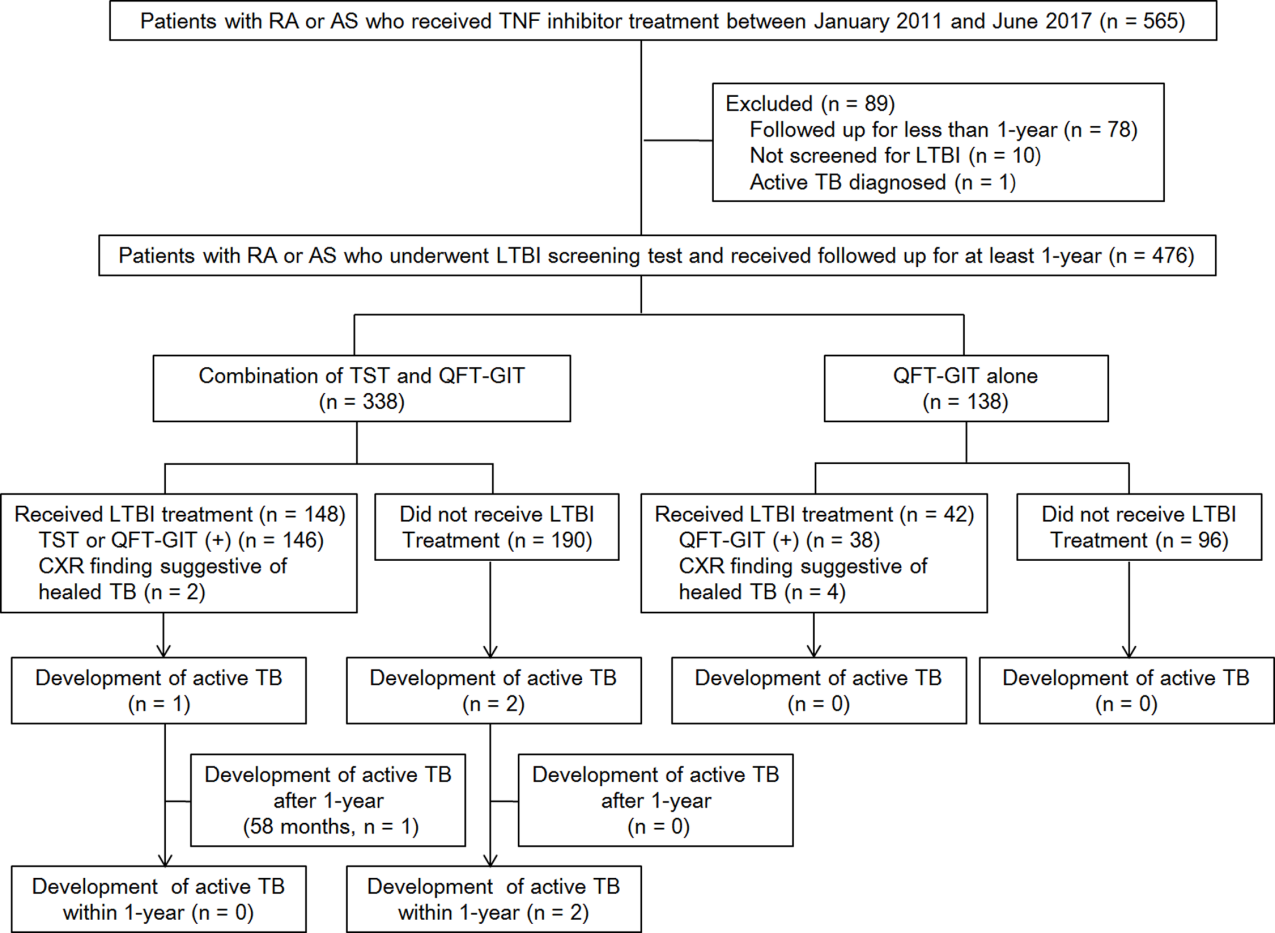
The study flowchart. RA, rheumatoid arthritis; AS, ankylosing spondylitis; TNF, tumor necrosis factor; LTBI, latent tuberculosis infection; TB, tuberculosis; TST, tuberculin skin test; QFT-GIT, QuantiFERON-TB Gold In-Tube; CXR, chest radiography.

Between January 2011 and June 2017, 565 patients with inflammatory arthritis, including RA or AS, received a TNF inhibitor at the Asan Medical Center (Seoul, South Korea). Of these 565 patients, we enrolled those who received LTBI screening tests and were followed up for ≥1 year after the TNF inhibitor initiation. The exclusion criteria were as follows: (i) patients who were followed up for <1 year; (ii) patients who did not receive an LTBI test; and (iii) patients who were diagnosed with active tuberculosis at the screening visit (Fig 1). We retrospectively reviewed the medical records of the remaining 476 patients in August 2017.

This study protocol was approved by the Institutional Review Board of Asan Medical Center (IRB No.: 2017–0014), which waived the requirement for informed consent because of the retrospective nature of the analysis.

### LTBI screening methods and screening strategy during the study period

During the screening visit, we registered information regarding the presence of any symptoms suggestive of tuberculosis, the presence of BCG scars, a medical history of previous tuberculosis treatment, and the use of disease-modifying anti-rheumatic drugs (DMARDs) or steroids. All patients underwent simple chest radiography, and those with radiographic abnormalities suggestive of tuberculosis were further assessed using sputum acid-fast bacilli smear and culture to rule out active tuberculosis or lung disease because of nontuberculous mycobacteria (NTM). The chest radiography was interpreted by a radiologist first and then independently interpreted by a pulmonologist again at the time of the screening visit. In case of discordant interpretations, they agreed on the chest radiography results after discussion. We performed TST using 2TU PPD RT23 (Statens Serum Institute, Copenhagen, Denmark) and the Mantoux approach. The induration size was measured after 48–72 h. In addition, the IGRA was performed using the QuantiFERON-TB Gold In-Tube (QFT-GIT; Cellestis, Carnegie, VIC, Australia) per the manufacturer’s instructions [18]; it was performed using whole blood collected into a heparin-containing tube, stimulated with negative control antigen (Nil), positive control antigen (Mitogen), or tuberculosis-specific antigens, and cultured for 16–24 h. We defined the TST induration of ≥10 mm as positive [19] and an interferon-γ value of ≥0.35 IU/mL as a positive QFT-GIT result. TST and QFT-GIT were performed on the same day.

The following strategies were used for LTBI screening during the study period: (i) from January 2011 to October 2014, the combination of TST and QFT-GIT (*n* = 337); (ii) between November 2014 and February 2015, QFT-GIT alone (*n* = 45) because of the temporary unavailability of PPD RT 23 in South Korea during this period; and (iii) since March 2015, either the combination of TST and QFT-GIT (*n* = 1) or QFT-GIT alone (*n* = 93) depending on the attending physician's choice. In patients who received LTBI screening with the combination of TST and QFT-GIT, we considered the LTBI as positive if either the TST or QFT-GIT result was positive. For patients with indeterminate QFT-GIT results, the presence of LTBI was determined by a combination of TST results and clinical factors including chest X-ray findings or previous history. We compared the screening strategies of QFT-GIT alone and in combination with TST.

### Criteria for commencing LTBI treatment and its regimen

We commenced LTBI treatment in patients with positive results of either TST or QFT-GIT, the absence of tuberculosis-suggestive symptoms, and normal chest radiography. In contrast, we did not commence LTBI treatment in patients with a history of previous adequate anti-tuberculosis treatment and no further tuberculosis exposure, despite positive TST and/or QFT-GIT result. Regardless of the TST or QFT-GIT results, patients without previous anti-tuberculosis treatment history who had abnormal chest X-ray findings suggestive of spontaneously healed tuberculosis, such as non-calcified nodules with distinct margins, discrete linear or reticular fibrotic scars, and fibrotic linear opacity, received LTBI treatment. In the time frame between November 2014 and February 2015 when PPD RT 23 was temporarily unavailable in South Korea, LTBI treatment was administered to all patients with indeterminate QFT-GIT. We initiated TNF inhibitor treatment 4 weeks after LTBI treatment.

During the study period, both isoniazid and rifampin for 3 months were the primary selected regimen for LTBI treatment [20]. In the case of adverse events or intolerance to isoniazid and rifampin in the 3-month regimen, we switched the regimen to either isoniazid for 9 months or rifampin for 4 months. We defined the treatment completion as taking >80% of all prescribed medication within 4 months for isoniazid and rifampin, 12 months for isoniazid, and 6 months for rifampin.

### Follow-up and the definition of active tuberculosis development

Irrespective of the LTBI status or treatment, all patients were asked to follow up by pulmonologists until 6 months after the completion of TNF inhibitor treatment. Usually, chest radiography was performed 3, 6, and 12 months after the initiation of TNF inhibitor treatment and every 12 months after that. In addition, all patients were instructed to visit their physicians if new symptoms or signs suggestive of tuberculosis developed. As the risk of tuberculosis development due to LTBI reactivation was highest within 1 year after the TNF inhibitor treatment commencement [21–23], only cases of tuberculosis development within 1 year were considered to be associated with the TNF inhibitor in this study if active tuberculosis developed during the follow-up period.

### Statistical analysis

We performed all analyses using SPSS software (version 20.0; SPSS, Chicago, IL). While the Student’s *t*-test was performed for continuous variables, the *χ*^2^ or Fisher’s exact test was performed for categorical variables. All tests of significance were two-sided, and we considered *P* < 0.05 as statistically significant.

## Results

### Clinical characteristics of the study cohort

In this study, 476 patients (mean age, 48.2 ± 14.9 years; 47% [227/476] males) with inflammatory arthritis who received LTBI test before the initiation of TNF inhibitor treatment fulfilled the eligibility criteria (Fig 1). All patients were tested for human immunodeficiency virus infection; all were negative. Inflammatory arthritis comprised RA in 266 (55.9%) and AS in 210 (44.1%) patients. During the study period, we administered four kinds of TNF inhibitors, and the most frequently prescribed TNF inhibitor was etanercept (34.7%, 164/476). Table 1 summarizes the detailed characteristics of the study cohort.

**Table 1:** Clinical Characteristics of 476 patients treated with TNF inhibitor according to the LTBI screening strategy.

Characteristics	Total (n = 476)	TST and QFT-GIT (n = 338)	QFT-GIT-alone (n = 138)	P—value
Age, years	48.2 ± 14.9	48.8 ± 15.4	46.6 ± 13.4	0.162
Male sex	227 (47.7)	166 (49.1)	61 (44.2)	0.383
Body mass index, kg/m^2^	24.2 ± 4.0	24.2 ± 4.0	24.3 ± 4.3	0.678
Current smoker	83 (17.4)	61 (18.0)	22 (15.9)	0.583
Inflammatory arthritis				0.779
Rheumatoid arthritis	266 (55.9)	187 (55.3)	79 (57.2)	
Ankylosing spondylitis	210 (44.1)	151 (44.7)	59 (42.8)	
Comorbidity				
Diabetes mellitus	21 (4.4)	16 (4.7)	5 (3.6)	0.772
Chronic lung disease^*^	28 (5.9)	21 (6.2)	7 (5.1)	0.791
Chronic kidney disease	3 (0.6)	2 (0.6)	1 (0.7)	0.868
Previous history of tuberculosis treatment	41 (8.6)	29 (8.6)	12 (8.7)	0.967
Positive BCG scar	305 (64.0)	215 (63.6)	90 (65.2)	0.740
CXR findings suggestive of healed TB	59 (12.4)	46 (13.7)	13 (9.4)	0.269
Anti-TNF agents used				0.030
Etanercept	164 (34.7)	127 (37.6)	37 (26.8)	
Adalimumab	162 (34.0)	109 (32.2)	53 (38.4)	
Infliximab	54 (11.3)	42 (12.4)	12 (8.7)	
Golimumab	96 (20.0)	60 (17.8)	36 (26.1)	
Sequential treatment of ≥2 TNF inhibitors	57 (12.0)	45 (13.3)	12 (8.7)	0.159
Anti-inflammatory drugs				
Steroid^†^	183 (38.4)	132 (39.1)	51 (37.0)	0.670
Methotrexate	221 (46.4)	151 (44.7)	70 (50.7)	0.230
DMARDs other than methotrexate	71 (14.9)	58 (17.2)	13 (9.4)	0.032
None	193 (40.5)	133 (39.3)	60 (43.5)	0.405

BCG, bacillus Calmette–Guérin; CXR, chest radiography; TB, tuberculosis; TNF, tumor necrosis factor; DMARD, disease-modifying anti-rheumatic drug; TST, tuberculin skin test; QFT-GIT, QuantiFERON-TB Gold In-Tube

^*^Chronic lung disease comprises interstitial lung disease (*n* = 16), bronchiectasis (*n* = 6), asthma (*n* = 4), chronic obstructive pulmonary disease (*n* = 1), and tuberculosis-destroyed lung (*n* = 1).

^†^The number of patients who received a prednisone dose se (drug; TST, tuberculin skin test; QFT-GIT, QuantiFERON-TB Gold In-TubeQFT-GIT, Quan.

Data are reported as mean ± standard deviations or number (%).

### LTBI screening methods and screening strategy

While 338 (71.0%) patients received LTBI test screening using the combination of TST and QFT-GIT, the remaining 138 (29.0%) received QFT-GIT-alone. We observed significant differences in the TNF inhibitor used and DMARDs other than methotrexate between patients who received the combination of TST and QFT-GIT strategy and QFT-GIT-alone. Table 1 summarizes the baseline characteristics of patients according to the LTBI screening strategy.

### LTBI test results and the percentages of patients identified for LTBI treatment

Among 338 patients who underwent LTBI test screening using the combination of TST and QFT-GIT strategy, the LTBI test results were positive in 159 (47.0%) patients; of these, 69 (43.4%) had positive results for both TST and QFT-GIT, 54 (34.0%) had positive TST and negative QFT-GIT results, 30 (18.9%) had negative TST and positive QFT-GIT results, and 3 (3.7%) had positive TST and indeterminate QFT-GIT results. The remaining 3 patients were diagnosed as having LTBI because their chest radiography findings were suggestive of spontaneously healed tuberculosis despite negative TST and IGRA results. Among 159 patients with positive LTBI results, 43.8% (148/338) required LTBI treatment. Of note, LTBI treatment was not required in the remaining 11 patients because of a history of previous adequate anti-tuberculosis treatment history and no further tuberculosis exposure despite positive QFT-GIT and/or TST results.

In contrast, the positive rate of LTBI test results and the percentage of patients who received LTBI treatment were both low among patients who underwent LTBI screening using the QFT-GIT-alone strategy. That is, 32.6% (45/138) of patients had positive LTBI results, and 30.4% (42/138) required LTBI treatment. The remaining 3 patients did not receive treatment because of a history of previous adequate anti-tuberculosis treatment and no further tuberculosis exposure. Table 2 presents the LTBI test results and the treatment rates of 476 patients according to the screening strategies.

**Table 2:** LTBI test results and percentages of patients who received LTBI treatment among 476 patients.

	TST and QFT-GIT (n = 338)	QFT-GIT-alone (n = 138)	P—value
Result of the LTBI test			0.005
Positive	159 (47.0%)	45 (32.6%)	
Negative	179 (53.0%)	93 (67.4%)	
LTBI treatment			0.007
Received	148 (43.8%)	42 (30.4%)	
Not received	190 (56.2%)	96 (69.6%)	

LTBI, latent tuberculosis infection; TST, tuberculin skin test; QFT-GIT, QuantiFERON-tuberculosis Gold In-Tube

Data are reported as number (%)

The completion rate of LTBI treatment with isoniazid and rifampin for 3 months was 87.4% (166/190). In the remaining 24 patients, we switched the regimen because of adverse events or intolerance. Among these 24 patients, 21 completed their treatment with the switched regimen; thus, the overall completion rate of LTBI treatment was 98.4% (187/190). The remaining 3 patients did not complete LTBI treatment (2 receiving the combination of TST and QFT-GIT and 1 receiving QFT-GIT-alone).

### Development of active tuberculosis

Of 476 patients, 2 (0.4%) developed active tuberculosis within 1 year after the initiation of TNF inhibitor. Table 3 presents the detailed characteristics of these 2 patients. Upon classifying the tuberculosis development according to the LTBI screening strategy, both cases of active tuberculosis were diagnosed among 338 patients who underwent LTBI test screening using the combination of TST and QFT-GIT (Fig 1). Among patients who underwent LTBI test screening using QFT-GIT-alone, active tuberculosis did not develop.

**Table 3:** Characteristics of 2 patients who developed active tuberculosis during TNF inhibitor treatment.

Characteristics	Patient 1	Patient 2
Age, year	56	62
Sex, M/F	Male	Male
Inflammatory arthritis	Ankylosing spondylitis	Rheumatoid arthritis
History of anti-tuberculosis treatment	No	No
Positive BCG scar	Unknown^*^	No
Baseline chest radiography	RUL 3mm nodule^†^	Normal
Baseline TST induration, mm	7	0
Baseline QFT-GIT, IU/mL	Negative (0.00)	Negative (-0.01)
LTBI treatment	No	No
TNF antagonist used	Adalimumab	Infliximab
Time to tuberculosis after the start of TNF antagonist, months	3.7	3.1
Sites of tuberculosis	Tuberculosis pleurisy	Miliary tuberculosis and tuberculosis pleurisy
Diagnostic method of tuberculosis	Culture-positive	Culture-positive
Drug susceptibility results	Pan-susceptible	Pan-susceptible
Outcome of anti-tuberculosis treatment	Cure	Cure
1-year recurrence after tuberculosis treatment	No	No
Reuse of TNF antagonist after anti-tuberculosis treatment	Yes	No

BCG, bacillus Calmette–Guérin; TST, tuberculin skin test; QFT-GIT, QuantiFERON-tuberculosis Gold In-Tube; LTBI, latent tuberculosis infection; TNF, tumor necrosis factor; TST, tuberculin skin test; QFT-GIT, QuantiFERON-tuberculosis Gold In-Tube; RUL, right upper lobe

^*^We did not determine the presence of BCG scar in patient 1. In addition, this patient and his family did not clearly remember the history of BCG vaccination.

^†^This lesion had not changed for 18 months on performing chest radiography before the TNF inhibitor initiation and was diagnosed as a focal calcified granuloma because of previous inflammation. Based on the Korean tuberculosis guidelines, LTBI treatment was not indicated for focal calcified granuloma. Furthermore, this lesion had not changed after tuberculosis pleurisy development.

## Discussion

Despite the increased use of a TNF inhibitor for inflammatory arthritis, optimal methods for LTBI screening in patients with inflammatory arthritis before the TNF inhibitor initiation remain controversial. Although several guidelines recommend (i) TST or IGRA-alone [10–12], or (ii) the combination use of TST and IGRA [24, 25] no study has compared these strategies directly. To the best of our knowledge, this is the first study to compare various LTBI screening methods, i.e., IGRA-alone versus the combination of TST and IGRA before the use of TNF inhibitor in patients with inflammatory arthritis. This study revealed that the number of patients who needed LTBI treatment was reduced among patients who received LTBI test using QFT-GIT-alone, compared with those who received LTBI test using the combination of TST and QFT-GIT. Besides, none developed active tuberculosis within 1 year after TNF inhibitor initiation among patients who received QFT-GIT-alone. These results suggest that QFT-GIT-alone could be used as a screening strategy for diagnosing LTBI in inflammatory arthritis patients in South Korea, where the incidence of tuberculosis is intermediate and BCG vaccination is mandatory at birth.

The findings of this study implied that, among patients screened for LTBI by using the combination of TST and IGRA, the discordant TST-positive and IGRA-negative (54/159, 34.0%) results were probably because of the false positivity for TST rather than to true LTBI; the most plausible explanation for this discrepancy is having obtained a previous BCG vaccination. In South Korea, infants are usually vaccinated with BCG at birth. In addition, before 1997, Koreans exhibiting a negative TST at 12 years of age were revaccinated with BCG; thus, those born before 1985 were revaccinated if the TST was negative at 12 years of age [26]. Although the Centers for Disease Control and Prevention recommendations state that the TST reactivity is unlikely to persist ≥10 years after BCG vaccination [27], some studies have reported that the TST positivity persists for ≥20 years [28–30]. A recent study provided evidence that BCG vaccination after infancy might affect TST results extending up to 55 years after vaccination [31]. Although we did not determine the presence of BCG scars in approximately one-third of patients in this study, we do not believe this implies that all patients had not received BCG vaccinations because the BCG vaccination does not always cause the characteristic raised scar [32]. Another study in South Korea reported that 30% of those born after 1985 had no BCG scar despite that approximately 90% of Koreans are administered the BCG vaccination because it is both free and mandatory [33], suggesting that the absence of a BCG scar is not a clear indication that an individual has never been vaccinated with BCG [26].

Another possible explanation for the discordant TST-positive and IGRA-negative results was the TST false positivity because of the exposure to NTM, which continues to increase around the world, including South Korea [34, 35]. Apparently, the results of TST are affected by the exposure to NTM, whereas a study reported that NTM did not affect the result of IGRA [36]. Indeed, we have previously reported that NTM infection is not rare, and infection rates are increasing in patients with RA at our center [37]. However, some NTM species, such as *Mycobacterium kansasii*, *Mycobacterium marinum*, and *Mycobacterium szulgai* can potentially cause a positive QFT-GIT result because these rare NTM species encode CFP-10 and ESTA-6, two antigens targeted by QFT-GIT [38, 39]; but, these NTM species, such as *M*. *kansasii*, are quite rare in South Korea [40, 41]. Irrespective of whether the cause of the TST false positivity was either BCG vaccination or NTM, the discordant TST-positive and IGRA-negative results because of false-positive TST could lower the specificity of LTBI diagnosis, which would then lead to increased costs, possible adverse effects in response to LTBI treatment, and delay in TNF inhibitor treatment [42].

Notably, guidelines differ regarding the optimal LTBI screening strategy before TNF inhibitor treatment. Some guidelines or experts endorse the recommendation that the combination of TST and IGRA should be used to determine the tuberculosis infection status of immunosuppressed patients, such as inflammatory arthritis, especially in regions where tuberculosis is prevalent (crude incidence ≥20/100,000 per year), to increase sensitivity [13, 14, 43]. Conversely, the American College of Rheumatology recommends the use of the IGRA over the TST in patients with RA who had previously received a BCG vaccination for screening to identify LTBI because of the high false-positive test rates for TST in these patients [10]. Some other guidelines propose similar recommendations; IGRA-alone could be performed in selected patients with a BCG history [5, 44]. We believe that our findings support the recommendation for the IGRA-alone strategy in patients with a BCG vaccination history.

Several studies have supported the use of the QFT-GIT-alone strategy as an LTBI screening method in patients with inflammatory arthritis. Another tertiary referral center in South Korea [23] used both TST and QFT-GIT for LTBI screening tests for patients with inflammatory arthritis, but only positive QFT-GIT results were considered an LTBI treatment indicator regardless of the TST results. They revealed that, among 60 patients who did not receive LTBI treatment with discordant TST-positive and IGRA-negative results, none developed active tuberculosis within 1 year after the TNF inhibitor initiation, thereby concluding QFT-GIT-alone might be used for diagnosing LTBI in countries such as South Korea. In Taiwan, before the initiation of biologics, LTBI was determined by IGRA-alone as per local guidelines because BCG is universally vaccinated in Taiwan [45]. A systematic review assessed three small longitudinal studies that enrolled patients screened for LTBI before starting TNF inhibitor in which none of the 29 patients with positive TST/negative IGRA results and none of the 146 patients with negative IGRA results, regardless of TST results, developed active tuberculosis after ≥12 months of follow-up [46]. However, none of these studies directly compared the combination of TST and QFT-GIT strategy with the QFT-GIT-alone strategy. The strength of our study is that it was the first to conduct such a comparison, which led to the finding that QFT-GIT-alone could be cautiously used for diagnosing LTBI in patients with inflammatory arthritis. However, it should be noted that the results of the present study did not allow us to identify which screening strategy is superior, because the two screening strategies were not randomly assigned and compared.

The limitations of this study must be considered to interpret our results correctly. Most importantly, since 2015, the choice of screening strategies either the combination of TST and QFT-GIT or QFT-GIT-alone was not randomized. Rather, it depended on the attending physician's discretion. Thus, it may seem that selection bias is inevitable. However, we believe that the impact of selection bias in this study might not have been profound because the number of patients who received the combination of TST and IGRA was only 1 of 94 patients who received TNF inhibitor since March 2015. In addition, we observed significant differences regarding the TNF inhibitor used and DMARDs other than methotrexate between patients who received the combination of the TST and QFT-GIT strategy and QFT-GIT-alone; this difference of medication could be attributed to the period when QFT-GIT-alone was primarily used in the latter period of the whole study duration. When we analyzed our data again after matching QFT-GIT-alone versus QFT-GIT and TST group by 1:1 ratio after controlling the medication, we did not observe significant differences (data not shown). Third, we did not confirm the adherence to treatment by directly observed treatment (DOT) in this study because Korean national tuberculosis guidelines have not yet adopted DOT. Although specially trained private–public mix cooperation (PPM) nurses monitored the treatment courses of our patients [17, 47], the possibility of noncompliance to LTBI treatment cannot be eliminated, which might have affected the LTBI treatment efficacy. Furthermore, the retrospective nature of the study may have limited the collection of accurate data regarding the close contact with active tuberculosis patients. Moreover, the most frequently prescribed TNF inhibitor of our study subjects was etanercept, which is known to have a lower risk of tuberculosis than anti-TNF monoclonal antibody. Fifth, although we concluded that the discordant TST-positive and IGRA-negative results were because of the BCG vaccination or the exposure to NTM, those results could have been because of true tuberculosis infection, as suggested previously [48–50]. Finally, considering that the number of patients who underwent LTBI screening test using QFT-GIT-alone as well as those who developed tuberculosis was insufficient for drawing a reliable conclusion, further extensive studies with larger numbers of patients are warranted to validate our findings.

In conclusion, this study suggests that QFT-GIT-alone could be used for diagnosing LTBI in patients with inflammatory arthritis before initiating TNF inhibitor in countries such as South Korea, where the tuberculosis prevalence is moderate and the BCG vaccination is mandatory at birth, rather than by using the combination of TST and QFT-GIT.

## Supporting information

S1 Dataset Raw data. (XLSX)
